# Supplementary material for: Association between oral microbiome and seven types of cancers in East Asian population: a two-sample Mendelian randomization analysis
Source: Front Mol Biosci. 2023 Nov 21;10:1327893. doi: 10.3389/fmolb.2023.1327893 (PMC10702768; doi:10.3389/fmolb.2023.1327893)
Supplement: Supplementary file 1 [file DataSheet1.ZIP › Supplementary Materials/MR plots of saliva/saliva═╝/colorectal cancer/pheno.3289_to_colorectal cancer_forest.pdf]

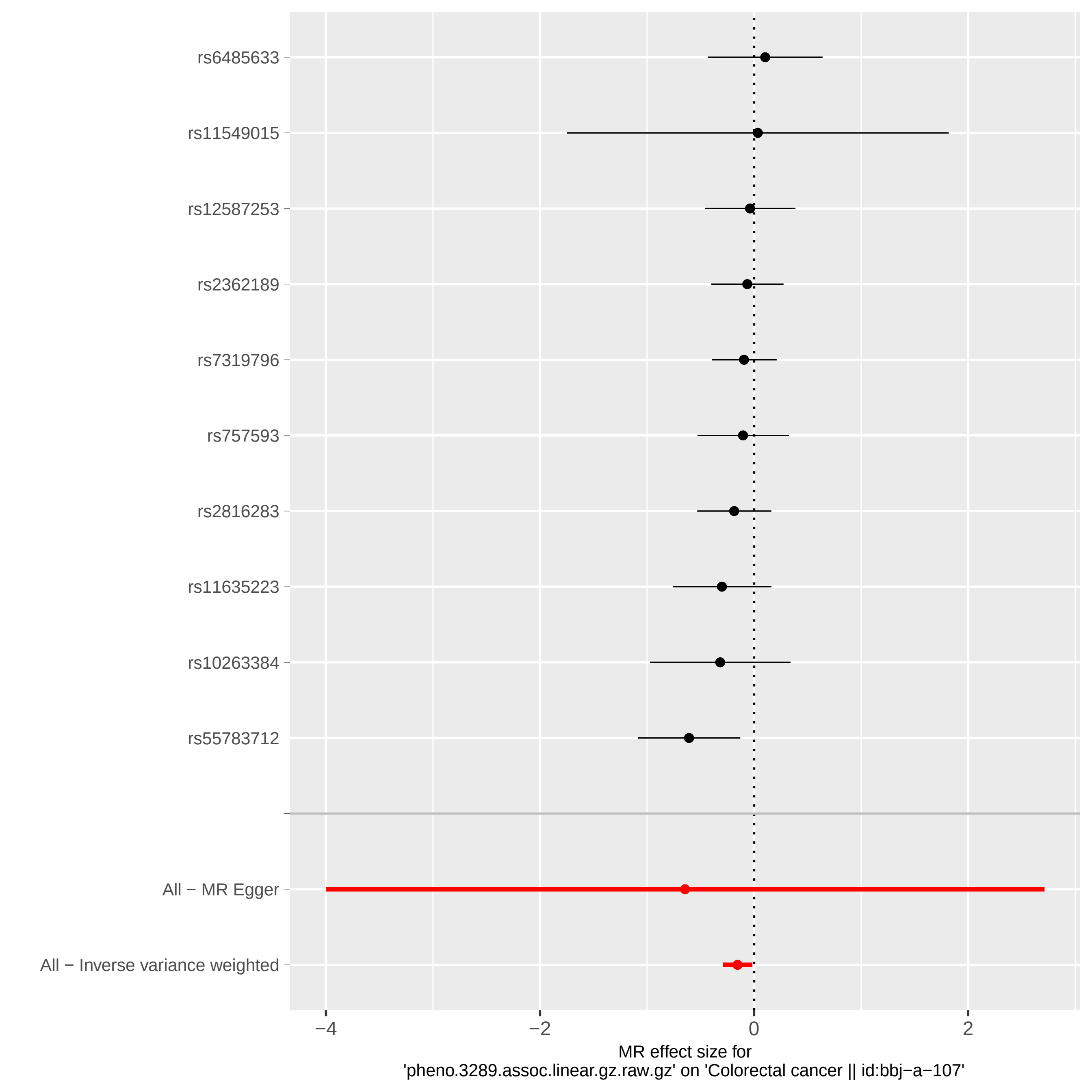
rs6485633
rs11549015
rs12587253
rs2362189
rs7319796
rs757593
rs2816283
rs11635223
rs10263384
rs55783712
All − MR Egger
All − Inverse variance weighted
−4
−2
0
2
MR effect size for
'pheno.3289.assoc.linear.gz.raw.gz' on 'Colorectal cancer || id:bbj−a−107'
